# Medical doctors’ awareness of radiation exposure in diagnostic radiology investigations in a South African academic institution

**DOI:** 10.4102/sajr.v23i1.1707

**Published:** 2019-04-30

**Authors:** Akingboye M. Dauda, John O. Ozoh, Olakunle A. Towobola

**Affiliations:** 1 Department of Diagnostic Radiology and Imaging, Dr George Mukhari Academic Hospital, Ga-Rankuwa, Pretoria, South Africa; 2 Department of Internal Medicine, School of Medicine, Sefako Makgatho Health Sciences University, Ga-Rankuwa, Pretoria, South Africa

**Keywords:** Awareness, medical doctors, diagnostic radiology, radiation protection, exposure, risk, patient safety, education and training

## Abstract

**Background:**

Diagnostic investigations using radiation have become a critical feature of medical practice in recent times. However, the possibility of doctors’ underestimation of risks of over-exposure of patients to diagnostic radiation still warrants further evaluation.

**Objectives:**

To investigate doctors’ awareness of diagnostic radiation exposure at Dr George Mukhari Academic Hospital, South Africa.

**Methods:**

This was a cross-sectional, analytical investigation of the awareness of doctors about radiation exposure in diagnostic radiology investigations. A cluster sampling technique was employed to recruit 217 participants. Consent and approval of the participants were sought and obtained before questionnaire administration during departmental meetings between October 2017 and March 2018.

**Results:**

Of the participants, 80% had no formal training on radiation exposure and 33.8% of them correctly estimated natural background radiation. Correct estimates of the effective dose from a single-view abdominal X-ray (AXR) were expressed by 7.5%, quantity of radiation of a single-phase computed tomography (CT) abdomen by 30.3% and dosage from a two-view unilateral mammogram by 29.1% of the participants. More than 75% of participants agreed that children are more sensitive to radiation, but only 10.5% suggested medical termination of pregnancy for a woman who had CT abdomen and pelvis with contrast. Dosage and risk of inducing fatal cancer from common but more complex imaging procedures were poorly understood. Only the doctors of the radiology department showed a statistically significant (*p* < 0.0001) association with regards to their radiation awareness.

**Conclusion:**

Because of the high rate of poor awareness of radiation risks observed in this study, it is important to initiate, early in the medical curriculum for medical students, the need for a rotation in the Department of Radiology, similar to such rotations in other medical specialties.

## Introduction

Medical diagnostic radiation has been the fastest growing source of human exposure to ionising radiation, with the collective radiation increased by a factor of six in the last two to three decades.^1^ Over the past 100 years, diagnostic investigations which use radiation have become a critical feature of standard medical practice.^2,3^ This may occur in successive rounds of diagnostic investigations to arrive at a diagnosis. In many cases, diagnostic investigations such as X-rays or mammograms are used to diagnose and treat a medical condition even before it is clinically apparent.^4^

This unbridled exposure to ionising radiation has been scientifically proven to cause damage to healthy tissues, such as skin burns and radiation sickness, at high exposures (deterministic effects) and also raises the risks of cancers and genetic damages (stochastic effects) at low exposures.^5^ Furthermore, errors in the radiation exposures that occur during diagnostic investigations usually go unrecognised or unreported and may be associated with high patient morbidity.

According to the 2007 International Commission on Radiological Protection recommendations,^6^ clinicians are expected to have full knowledge of potential benefits and detriments associated with medical radiation exposure in order to justify exposure. However, the report by Holmberg et al.^7^ showed that awareness of doctors about radiation exposure and associated cancer risk is poor. While the epidemiological data suggested that ionising radiation levels as low as 50 millisieverts (mSv) have been implicated in the development of solid tumors,^6^ some recent surveys illustrated that radiation dose is not an important consideration for clinicians when they refer their patients for diagnostic radiological examinations.^7^ Hence, doctors tend to underestimate the risks of patients’ exposure to diagnostic radiation.^8^ Many physicians have been reported to have little or no training on radiation protection, while many have no qualified medical physicists’ support.^9^

The importance of these findings lies in the fact that when doctors have poor awareness of radiation risks, inherent in diagnostic radiology examinations, they will not be able to counsel their patients and request for appropriate examinations based on the principle of benefits outweighing the risks. This is much more important especially in paediatric patients in whom radiation must be kept to the barest minimum if not possible to avoid altogether. This is because their tissues are highly radiosensitive. In addition, children will also live longer and are more likely than adults to develop radiation-induced cancer. Also, as future parents, they are at risk of passing on radiation-induced genetic defects to the next generation.

Education of medical professionals in radiation protection issues and radiation safety has been a continuous problem even in well-developed countries.^10^ This study therefore investigated doctors’ awareness of diagnostic radiation exposure at Dr George Mukhari Academic Hospital (DGMAH), South Africa.

## Research methodology

### Study design, setting and population

A cross-sectional analytical method was used to explore the awareness of practising doctors employed by DGMAH about radiation exposure in diagnostic radiology. A cluster sampling technique was applied to recruit 217 volunteer participants in this study, after assuring them of the confidentiality of information supplied. The consent and approval of the heads of each department in the hospital were sought and obtained before questionnaire administration, which was carried out during departmental meetings from October 2017 to March 2018. Each department represented a cluster and being a single-stage cluster sampling, questionnaires were distributed at the same time to all doctors, including consultants, registrars, medical officers and interns present in each cluster meeting.

The inclusion criteria entailed that respondents were practising doctors, accredited as medical practitioners by the Health Professions Council of South Africa (HPCSA) and were employed by DGMAH. Professionals who were not clinicians or doctors employed by DGMAH, but present at each meeting, were excluded from this study.

#### Instrument

The assessment tool was a self-reported questionnaire which was developed from three previously published studies.^11,12,13^ The questionnaire consisted of 26 questions relating to doctors’ awareness of exposure to diagnostic radiation.

### Data collection and analysis

A total of 217 doctors were enrolled in the study. The questionnaires were individually handed over to the doctors and collected by the researcher. Respondents who had been interviewed earlier were exempted from the subsequent data collection. For awareness scoring, one positive point was awarded for each correct answer. In addition, according to the total number of items, 0 was regarded as minimum score and the maximum overall score was 26. Scores less than 50% were considered as poor, those between 50% and 75% were considered as fair, while greater than 75% was considered as good awareness. Mann–Whitney and Kruskal–Wallis tests were used to compare the responses among groups. The characteristics of the participants were obtained through descriptive analysis using frequencies and percentages, while Fisher’s exact test was used to find out the association between doctors’ demographic characteristics and their awareness of diagnostic radiation exposure. A *p*-value of ≤ 0.05 was considered as a cut-off point for significance. The data were managed and analysed by using SPSS version 20.0 (IBM, New York City, USA).

### Bias

A potential information bias may involve over-reporting of diagnostic X-ray procedures. This could possibly arise from the participants who may be more conscious of this type of exposure and therefore may have put more individual effort into recalling their diagnostic X-ray procedures when filling out the questionnaire. Selection of participants from different departments within the hospitals using cluster sampling was expected to significantly minimise this bias.

## Ethical consideration

Ethical approval was obtained from Sefako Makgatho Health Sciences University Research Ethics Committee (SMUREC) before the commencement of this study (SMUREC/M/203/2017:PG). Approval was also obtained from the chief executive officer (CEO) of Dr George Mukhari Academic Hospital. Confidentiality of the participants was maintained as the names and other identifications were not required during the data collection process.

## Results

The investigations into medical doctors’ awareness of exposure to diagnostic radiation showed that more than 80% had not had any formal training on radiation exposure. Only 33.8% of doctors correctly estimated the average natural background radiation. Furthermore, the comparison of the radiation dose from a chest X-ray (CXR) to the annual dose a person receives from background radiation (1/10) was only correctly estimated by 20.6%, while the quantity of radiation a patient absorbs during a CXR (0.02 mSv) was only correctly estimated by 14.7% of respondents. The effective dose received by a patient in a two-view CXR was correctly estimated by the majority of doctors (54.6%) as twice the single-view CXR dose (Table 1).

**TABLE 1:** Doctors’ awareness of exposure to diagnostic radiation.

Parameters	Frequency	%
**Respondents ever had any formal training about ionising radiation (*n* = 200)**		
Yes	36	18
No	164	82
**Average natural background radiation is in the range (*n* = 145)**		
20–30 mSv	39	26.9
2–3 mSv†	49	33.8
0.2–0.3 mSv	46	31.7
200–300 mSv	11	7.59
**Comparison of the radiation dose from a chest X-ray to the annual dose a person receives from background radiation (*n* = 165)**		
1/100	47	28.5
1/10†	34	20.6
Equal	24	14.6
10 times	39	23.6
100 times	21	12.73
**Quantity of radiation a patient absorbs during a chest X-ray (*n* = 156)**		
0.02 mSv†	23	14.7
0.2 mSv	54	34.6
2 mSv	35	22.4
20 mSv	37	23.7
200 mSv	7	4.5
**Approximate effective dose received by a patient in a two-view chest X-ray is (*n* = 154)**		
Almost equal to single-view chest X-ray	27	17.5
Twice the single-view chest X-ray†	84	54.6
Five times the single-view chest X-ray	21	13.6
10 times the single-view chest X-ray	22	14.3

mSv, millisieverts.

† , Correct responses.

Almost half of the doctors in this study showed that effective dose from a single-view abdominal X-ray (AXR) is equivalent to 1–10 CXR and that computed tomography (CT) abdomen single phase gives a dose of 100 mSv. Dosage from a two-view unilateral mammogram was stated to be almost equal to a single-view CXR by 38.8% doctors (Table 2).

**TABLE 2:** Doctors’ awareness of diagnostic radiation doses.

Parameters	Frequency	%
**Effective dose from a single-view AXR is equivalent to (*n* = 160)**		
0–1 CXR	39	24.4
1–10 CXR	74	46.3
10–50 CXR	35	21.9
50–100 CXR†	12	7.5
**CT abdomen single phase gives a dose of (*n* = 162)**		
10 mSv†	49	30.3
100 mSv	76	46.9
1 mSv	19	11.7
None	18	11.1
**Dosage from a two-view unilateral mammogram is (*n* = 165)**		
Almost equal to single-view chest X-ray	64	38.8
Twice the single-view chest X-ray†	48	29.1
10–20 times the single-view chest X-ray	41	24.9
50–100 times the single-view chest X-ray	12	7.3

CT, computed tomography; CXR, chest X-ray; mSv, millisieverts.

† , Correct responses.

**FIGURE 1:**
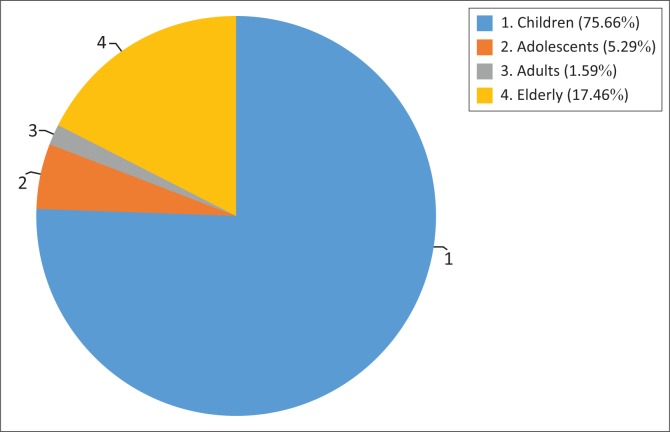
Respondents’ perceptions of the category of people most sensitive to radiation (*n* = 189).

More than 75% of doctors considered children as the most sensitive to radiation, while less than 20% perceived that the elderly were the best suited for this category (Figure 1).

**FIGURE 2:**
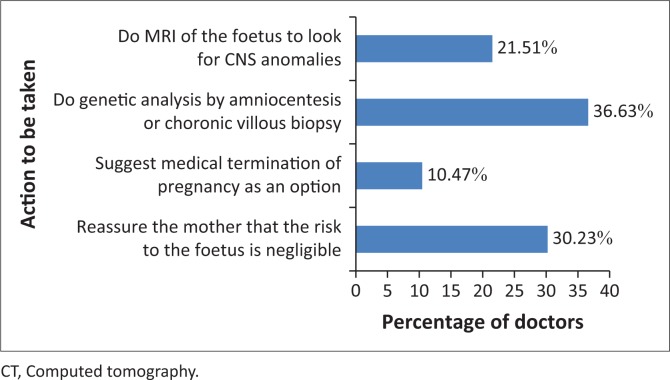
Action to be taken according to American College of Radiology guidelines in a case of a pregnant woman that underwent computed tomography abdomen and pelvis with contrast, as her pregnancy status was not enquired into by the computed tomography technologist before performing computed tomography (*n* = 170).

In line with the American College of Radiology (ACR) guidelines, actions mostly recommended by the respondents in a situation where a pregnant woman had already undergone CT abdomen and pelvis with contrast without the radiologist’s knowledge of her pregnancy were to conduct a genetic analysis by amniocentesis or chorionic villous biopsy (36.6%) or to reassure the mother that the risk to the foetus is negligible (30.2%) (Figure 2).

The most absorbed X-ray units as quantified by the respondents were between 1 and 10 units for AXR (39.8%), intravenous urography (IVU) (39.4%), barium enema (30.9%) and ventilation/perfusion scan (V/Q) (31.8%). Ultrasound (US) of the abdomen (64.2%), magnetic resonance imaging (MRI) of the brain (non-contrast) (48.5%) and MRI of the brain with intravenous (IV) contrast (35.5%) were considered to absorb between 0 and 1 units of X-ray. CT abdomen (with IV contrast) was mostly stated to absorb 50–100 units (26.9%) of X-ray (Table 3).

**TABLE 3:** The numbers of doctors who knew the doses of common but more complex imaging procedures.

Type of investigations	0–1 u		1–10 u>		10–50 u		50–100 u		100–500 u		Total	
	*n*	%	*n*	%	*n*	%	*n*	%	*n*	%	*n*	%
Abdominal X-ray	65	36.9	70	39.8	37	21.0†	3	1.7	1	0.6	176	100
IVU	31	18.8	65	39.4	53	32.1	15	9.1	1	0.6†	165	100
Barium enema	46	27.4	52	30.9	49	29.2	17	10.1	4	2.4†	168	100
Abdominal ultrasound	104	64.2†	29	17.9	19	11.7	10	6.2	0	0	162	100
Brain MRI (non-contrast)	83	48.5†	31	18.1	21	12.3	23	13.5	13	7.6	171	100
Brain MRI with IV contrast	61	35.5†	47	27.3	24	13.9	23	13.4	17	9.9	172	100
V/Q scan	31	18.2	54	31.8	41	24.1	33	19.4†	11	6.5	170	100
CT abdomen (with IV contrast)	15	8.8	40	23.4	40	23.4	46	26.9	30	17.5†	171	100

CT, computed tomography; IV, intravenous; IVU, intravenous urography; US, ultrasound; MRI, magnetic resonance imaging; V/Q scan, ventilation/perfusion scan.

Note: Take a chest X-ray count as 1 unit (u).

† , Correct responses.

The risk of inducing a fatal cancer from the radiation absorbed during diagnostic investigations was, in most instances, quantified as less than 1 in a million for AXR (55.8%), IVU (42.7%), barium enema (48.2%), abdominal ultrasound (75.3%), brain MRI (non-contrast) (60.5%), brain MRI with IV contrast (42.8%) and V/Q scan (32.5%). The risk associated with CT abdomen (with IV contrast) was assessed as 1 in 300 000–1 in 10 000 (32.3%) (Table 4).

**TABLE 4:** The number of doctors who knew the risk of inducing fatal cancer from common but more complex imaging procedures.

Type of investigations	Less than 1 in a million		1 in a million to 1 in 300 000		1 in 300 000 to 1 in 10 000		1 in 10 000 to 1 in 5000		1 in 5000 to 1 in 1000		Total	
	*n*	%	*n*	%	*n*	%	*n*	%	*n*	%	*n*	%
Abdominal X-ray	96	55.8	36	20.9	25	14.5†	11	6.4	4	2.3	172	100
IVU	70	42.7	54	32.9	29	17.7	8	4.9†	3	1.8	164	100
Barium enema	80	48.2	47	28.3	28	16.7	9	5.4	2	1.2†	166	100
Abdominal ultrasound	122	75.3†	24	14.8	8	4.9	4	2.5	4	2.5	162	100
Brain MRI (non-contrast)	98	60.5†	28	17.3	23	14.2	9	5.6	4	2.5	162	100
Brain MRI with IV contrast	68	42.8†	47	29.6	25	15.7	11	6.9	8	5.0	159	100
V/Q scan	53	32.5	45	27.6	38	23.2†	17	10.4	10	6.1	163	100
CT abdomen (with IV contrast)	27	16.5	39	23.8	53	32.3	22	13.4	23	14.0†	164	100

CT, computed tomography; IV, intravenous; MRI, magnetic resonance imaging; IVU, intravenous urography; V/Q scan, ventilation/perfusion scan.

† , Correct responses.

**FIGURE 3:**
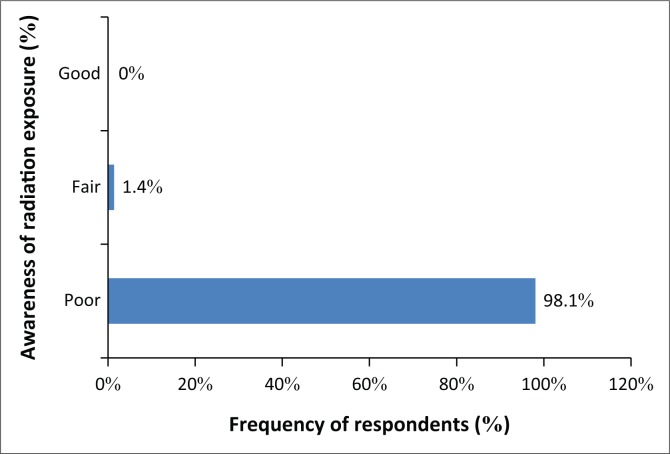
Respondents’ awareness of risk in diagnostic radiation.

**FIGURE 4:**
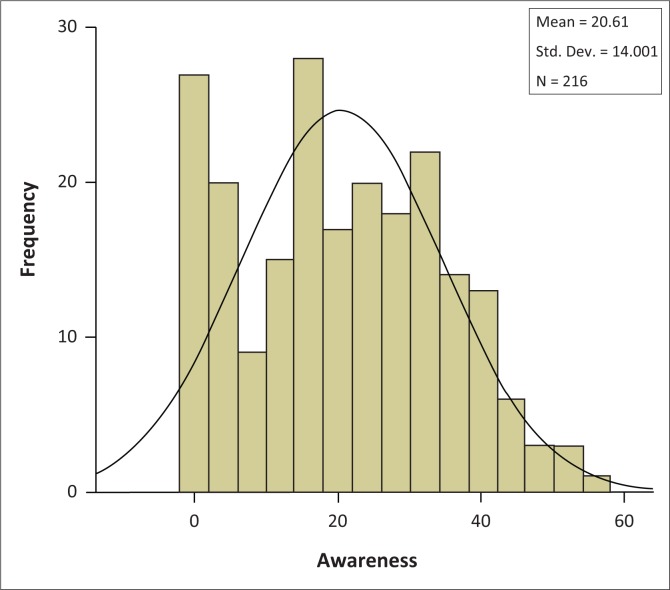
Distribution of respondents based on their awareness of exposure to radiation (*n* = 216).

Nearly all the medical doctors in this study (98.10%) demonstrated poor awareness of radiation risks, and only 1.40% expressed fair awareness, while none showed good awareness (Figure 3). The scoring of doctors’ awareness of radiation risk was normally distributed with mean and standard deviation value of 20.16 ± 14.0 (Figure 4).

The association between respondents’ awareness of exposure to diagnostic radiation and socio-demographic variables was not significant for most of the variables except the respondents’ departments which showed a statistically significant effect (*p* < 0.0001) (Table 5). This indicated that a higher proportion of doctors in the radiology department were more likely to have good awareness of exposure to diagnostic radiation than doctors from other departments.

**TABLE 5:** Association between respondents’ socio-demographic characteristics and their awareness of exposure to diagnostic radiation.

Parameters	Variables	Awareness						Test of significance		

		Poor		Fair		Total		Fisher’s exact test	*df*	*p*
		*n*	<50%	*n*	50% – 75%	*n*	%			
Gender	Male	133	99.3	1	0.8	134	100	3.71	2	0.563
	Female	78	97.5	2	2.5	80	100			
	**Total**	**211**	**98.6**	**3**	**1.4**	**214**	**100**			
Age (years)	≤ 30	47	100	0	0	47	100	2.97	4	0.429
	31–40	97	99	1	1.0	98	100			
	41–50	52	96.3	2	3.7	54	100			
	51–60	7	100	0	0	7	100			
	> 60	5	100	0	0	5	100			
	**Total**	**208**	**98.6**	**3**	**1.4**	**211**	**100**			
Years of clinical practice	< 5	58	98.3	1	1.7	59	100	0.976	3	1.00
	5–10	80	98.8	1	1.2	81	100			
	11–20	48	98	1	2.0	49	100			
	>20	17	100	0	0	17	100			
	**Total**	**203**	**98.5**	**3**	**1.5**	**206**	**100**			
Employment level	Interns	43	100	0	0	43	100	4.139	4	0.691
	Medical officers	36	97.3	1	2.7	37	100			
	Resident doctors	83	97.7	2	2.4	85	100			
	Consultants	38	100	0	0	38	100			
	**Total**	**200**	**98.5**	**3**	**1.5**	**203**	**100**			
Department of the participants	Anaesthesiology	50	100	0	0	50	100	20.4	10	0.0001
	Gynaecology and Obstetrics	34	100	0	0	34	100			
	Internal Medicine	31	100	0	0	31	100			
	Orthopaedics	6	100	0	0	6	100			
	Surgery	5	100	0	0	5	100			
	Emergency Unit	5	100	0	0	5	100			
	Paediatrics	12	100	0	0	12	100			
	Radiology	6	66.7	3	33.3	9	100			
	Family Medicine	31	100	0	0	31	100			
	Urology	5	100	0	0	5	100			
	Ophthalmology	7	100	0	0	7	100			
	**Total**	**192**	**98.5**	**3**	**1.5**	**195**	**100**			

## Discussion

Investigations into the awareness of doctors about diagnostic radiation exposure revealed that over 80% of them have never had any formal training about ionising radiation. This possibly explains the poor awareness of most of the physicians about routine radiology examinations, as observed in the report of Azmoonfar et al.^13^ The low number of respondents that demonstrated awareness of the average natural background radiation as 2 mSv–3 mSv and the quantity of radiation a patient absorbs during a CXR (0.02 mSv) affirmed an earlier report.^14^ Furthermore, Mahesh in his report compared the radiation exposure from one CXR as equivalent to the amount of radiation exposure one experiences from the natural surroundings in 10 days,^15^ which was also in line with the understanding of 20.61% respondents. In addition, the approximate effective dose received by a patient in a two-view CXR is considered twice the single-view CXR,^15,16^ as rightly indicated by the majority of the respondents.

An estimated exposure range of approximately 10–20 mSv per procedure, depending on the type of imaging test, and multiple tests have been reported to result in cumulative exposures of more than 100 mSv.^17^ This was similar to the effective dose from a single-view AXR correctly recorded by 7.5% of doctors as 50–100 CXRs. Although most of the respondents in this study evaluated the CT abdomen single phase as a dose of 100 mSv (46.91%), the correct response of 10 mSv radiation dose by over 30% respondents corroborated the report of abdomen and pelvis scans of a routine CT without contrast that had the lowest median effective dose of 15 mSv (interquartile range 10 mSv–20 mSv). The respondents also reported that a multiphase abdominal and pelvis CT had the highest median effective dose of 31 mSv (interquartile range of 21 mSv–43 mSv).^16^

Generally, dosage from a two-view unilateral mammogram was said to be almost equal (38.8%), twice (29.1%), 10–20 times (24.9%) or 50–100 times (7.3%) to single-view chest X-ray, respectively. However, the record of children being the category of people most sensitive to radiation as indicated by more than 75% participants in this study is in line with the findings of Ramanathan and Ryan.^18^ Furthermore, the doctors gave varying responses for the action to be taken (based on ACR guidelines) in the case of a pregnant woman who underwent CT abdomen and pelvis with contrast because the pregnancy status was not enquired into by the CT technologist before performing CT. Only 10.5% of the doctors gave the correct response of offering the option of medical termination of pregnancy – a view which was rightly supported by a previous study.^19^ While exposure to less than 50 mGy has not been associated with an increase in foetal anomalies or pregnancy loss, foetal radiation doses greater than 50 mGy can produce a subsequent increase in the risk of childhood cancer,^19^ coupled with initial risks such as abortion (15%), congenital anomalies (3% – 5%), intrauterine growth retardation (4%) and mental retardation (1%) that are always present in the pregnancy of every healthy woman.^20^

Except in abdominal ultrasound investigations where more than 64% respondents displayed good awareness of the recommended doses of imaging procedure, only a small percentage of the doctors demonstrated good awareness of radiation doses in other investigations such as AXR (21.02%), V/Q scan (19.41%), IVU (0.61%), barium enema (2.3%), CT abdomen (17.54%), brain MRI (non-contrast) (48.54%) and brain MRI with IV contrast (35.47%). This is in contrast to a survey conducted in Northern Ireland, where an improved awareness of the doctors in comparison with the result of the present study was attributed to the formal training about ionising radiation.^11^ However, the findings of this study are consistent with the observation of Shialkar et al.,^21^ who reported that 97% of physicians studied were not aware of the radiation doses received by patients during radiological investigations. A similar observation was also recorded for the number of doctors who knew the risk of inducing fatal cancer from common but more complex imaging procedures. While records of good awareness of radiation risk in investigations, such as abdominal ultrasound (75.31%) and brain MRI (non-contrast) (60.49%), were observed among the respondents, their awareness of risk in other investigations evaluated was not encouraging.^20,22^

Generally, the results of 98.10% of doctors who displayed poor awareness of radiation exposure in diagnostic radiology investigations corroborated the findings of some earlier investigations.^23,24,25^

Despite the higher number of male doctors compared to female doctors who participated in this study, female doctors displayed a higher level of awareness than the male doctors. Although the difference was not significant, this gender distribution was found to be in line with the finding from a study by Kamble et al.^25^

Only participants from the radiology department demonstrated fair awareness of radiation exposure in diagnostic imaging, and this could be associated with the earlier training and ethics of their job.^26^ To a large extent, the department from which the doctors came significantly impacted their awareness of radiation exposure.^27,28,29^ Therefore, it is advisable to put more emphasis on diagnostic radiation courses and education, as well as justification of referral for imaging among clinicians of all specialities at graduate and postgraduate levels.

The present study has some limitations. Firstly, it was conducted as a single-centre study at DGMAH, which prevented the researcher from making comparisons and eventually finding out any differences among doctors from different academic institutions. While this could be an interesting extension of the present work that might warrant further investigation, it was noted that the teaching curricula for doctors in the whole of South Africa are harmonised by the Department of Higher Education and Training (DHET) and the Colleges of Medicine of South Africa (CMSA). This harmonisation should contribute to mitigating potential inter-institutional discrepancies. Secondly, the sample size was not large compared with other studies,^22,31^ but can be deemed to be of average size, similar to a few other studies,^13,30,32,33^ and was sufficient to make statistically significant conclusions and correlations among doctors.

Based on the findings of this study, the authors would recommend the following:

- The management of DGMAH needs to conduct regular refresher programmes for its doctors on radiology investigations, with the aim of ensuring their adequate knowledge and awareness of radiation exposure in diagnostic radiology investigations.
- The management of DGMAH and the Department of Health (DoH) must ensure that appropriate radiological examination guidelines are drafted, vigorously circulated, put into practice and enforced at all levels of healthcare services.
- It is advisable for the DHET, DoH and CMSA to put more emphasis on the diagnostic radiation courses and education at graduate and postgraduate levels, as well as justification of referral for imaging among clinicians of all specialities. Medical students should be exposed to the Department of Radiology as much as they rotate in other medical specialities such as internal medicine and surgery.
- Internship rotations should be made to include compulsory rotations in the Department of Diagnostic Radiology.

These will be achieved by doing the following: Efforts will be made to give the recommendations to the management of DGMAH and seek an audience to discuss the findings, implications and recommendations. Secondly, with the permission of the DGMAH’s management, the authors will write a memorandum relating to the findings of this research and recommendations to the DoH, DHET, CMSA and HPCSA.

## Conclusion

The value of radiation and various technologies that utilise radiation are undisputed. However, the enormous growth in their use has not been paralleled by adequate education about the associated doses and risks of diagnostic ionising radiation. The results of this survey confirm that awareness of diagnostic radiation and its associated cancer-causing risks are inadequate across the medical professions. In certain clinical settings, doctors with inadequate awareness of these issues may be unable to perform required risk–benefit analyses and, therefore, will be incapable of fully informing their patients about these issues.

Injudicious use of diagnostic radiation is a small but concerning feature of modern medicine, and such practices may result in unnecessary exposure, avoidable stochastic effects, medico-legal uncertainty and, in some cases, an abandonment of evidence-based medicine. Improved education about radiation doses and potential risks from imaging is necessary across all levels of medical professions to ensure optimal use of these important diagnostic tools and the preservation of best medical practices. The educational programme for residents needs to focus not only on image interpretation but also on radiation awareness. Focussing on these aspects will be an important move towards minimising wastage of resources, reducing unnecessary radiation exposure and improving patient safety.
